# Disrupting the cycle of youth violence: The role of social support for youth in a Northern Irish Youth Work Programme

**DOI:** 10.1007/s40653-023-00529-x

**Published:** 2023-03-28

**Authors:** Colm Walsh

**Affiliations:** Queen’s University Belfast, Belfast, Northern Ireland

**Keywords:** Adversity, Psychological Stress, PTSD, Prevention, Social Support, Trauma, Youth Violence, Youth Work

## Abstract

Youth violence is a significant concern and previous research has found that violence is both trauma inducing and violence inducing. Meta-analyses have demonstrated that peri-trauma contextual factors such as the presence or absence of social supports following the onset of trauma may be predictive of the onset and duration of psychological stress. The aim of this study is to build upon the existing research evidence to clarify the links between social support, psychological stress and physical violence among a cross-section of youth living in high-violence areas of Northern Ireland. Participants were a sample of 10–25-year-olds (*N* = 635) who participated in a targeted youth work programme in Northern Ireland. This study conducted a mediation analysis, entering social support as the independent variable, psychological distress as the mediator and self-reported violence as the outcome variable. Violent victimisation was entered as a covariate in the analysis*.* After controlling for violent victimisation, social support operates through psychological stress to influence the risk of physical violence. Social support may contribute to reductions in psychological stress and thus buffer against the risks of living in areas of elevated community violence. Specialist youth work approaches may provide an opportunity to reduce psychological stress and thus help to mitigate the risk of further violence. Combined, these insights provide opportunities for harm reduction and prevention. At the same time, these findings advance our understanding of the distinct mechanisms of change involved in youth work-led violence prevention efforts.

## Introduction

### The Issue of Youth Violence

Interpersonal violence is a global concern. There are an estimated 500,000 people murdered each year and this is increasing at an annual rate of 2% (Mitis & Sethi, 2015), thus contributing to enduring concerns regarding its frequency, intensity and impact (Walsh, 2021; Walsh & Smyth, 2022; YEF, 2022). Youth violence has received particular policy attention over recent years (YEF, 2022), and whilst temporary reductions in youth violence were observed during the Covid-19 pandemic (Ellis et al., 2021; Reid & Baglivio, 2022), incidences in Northern Ireland appear to have returned to, and even exceeded, pre-pandemic levels (PSNI, 2022)—an observation that is consistent with wider empirical findings (Ellis et al., 2021; Reid & Baglivio, 2022).

### Clusters of Harm

Despite the signs that violence is increasing, it is likely that exposure to violence is highly heterogenous. Previous studies suggest that not all youth are at the same level of risk (Silvern & Griese, 2012; YEF, 2022), and not all youth are at elevated risk of the onset of violence-related harms following exposure. Factors such as being justice involved and being care experienced (Dierkhising et al., 2013); living in areas of higher ecological stress (Nygaard et al., 2018); and/or living in conflict affected areas (Haj-Yahia et al., 2021) make some clusters of young people more vulnerable to the traumatic effects of community violence than others. It is within these clusters that youth are more likely to report more frequent and varied exposure to violent adversity and also meet the threshold for diagnosable mental health conditions such as Post-Traumatic Stress Disorder (PTSD).

In Northern Ireland, where the legacy of violent conflict endures (DOJ, 2022), Redican et al. (2022) highlighted how a relatively large proportion of the general youth population have been exposed to multiple, and also co-occurring incidences of violence. In the first population-based well-being survey capturing the needs of Northern Irish youth, Bunting et al. (2020) established that violence was the single most commonly experienced form of childhood adversity. Indeed, 9% of this representative sample had experienced violent victimisation in the community. Estimates also suggest that mental health issues are also elevated in Northern Ireland with 13% experiencing any mood disorder and 2% experiencing symptoms consistent with psychological stress disorders such as PTSD. Cross-sectional studies have estimated that these rates could be even higher in those areas characterised as having greater concentrations of violence. For example, a study by Walsh (2021) found that across a sample of more than 400 Northern Irish youth, the rate of exposure to community violence was almost four-times higher than the population estimate, and that the rate of probable PTSD was eight-times higher than the population estimate.

### Adversity, Elevated Psychological Stress and Violence

PTSD is a complex and sometimes chronic mental health disorder that causes substantial distress and may interfere with social, emotional, behavioural and educational functioning (Trickey et al., 2010; Malvaso et al., 2022). Interpersonal adversities also appear to be particularly salient in regard to the onset of psychological stress, and clinically diagnosable disorders such as PTSD (Finkelor et al., 2007; Leenarts et al., 2013; Nöthling et al., 2019; Hamby et al., 2021; Zalta et al., 2021), with exposure to community violence particularly associated with elevated stress responses (Buka et al., 2001; Gaylord-Harden et al., 2017). There also appears to be a dose–response effect wherein those exposed to a greater number and type of adversity are at elevated risk of psychopathology. A body of evidence has identified that a link exists between traumatic distress, mental health disorders and disturbances in behavioural and emotional regulatory systems (see for example, Bremner & Vermetten, 2001; Fowler et al., 2009), that may in context, elevate the risk of further violence (Widom, 1989; Ardino, 2012; Baglivio et al., 2021; Malvaso et al., 2022; Walsh et al., 2021). This is a well-established criminological observation often coined as the *‘cycle of violence’* (Widom, 1989) or victim-perpetrator overlap (Wright et al., 2019). Understanding this association is vital for harm reduction and violence prevention.

### Social Support and Psychological Stress

Whilst the aetiology of psychological distress following violent trauma is not well established (Maschi & Bradley, 2008), several meta-analyses have demonstrated that peri-trauma contextual factors (Sperry & Widom, 2013) such as the presence or absence of social supports may remediate or elevate distress in adults (Brewin et al., 2000; Ozer et al., 2003; Blais et al., 2021) as well as in youth (Trickey et al., 2010; Sperry & Widom, 2013). Despite definitional difficulties (Sperry & Widom, 2013; Hansford & Jobson, 2022), social support can be defined as *"… the assistance and protection given to others"* (Langford et al., 1997: 95) and in his address to the Academy of Criminal Justice Sciences, Francis Cullen claimed that social support should be an organising framework for all criminological research. Cullen (1994) pointed to the many observations of the inverse relationship between social support and crime. Social support can be conceived as the functional and structural resources that are available to individuals, with the former capturing resources within a person’s social network (Wagner et al., 2016), and the latter referring to the size, availability and complexity of that social network (Guay et al., 2011).

Given what we already know regarding the association between psychological stress and violent outcomes, psychological stress can be hypothesised to be causally related to perceived rates of social support and thus predict outcomes such as violence. If empirically supported, social support as an organising construct has the potential of reducing the risk of violence through the mechanism of psychological stress reduction-a hypothesis consistent with mainstream criminological theory (Agnew, 2001; Zalta et al., 2021).

Social support may act as a buffer against the debilitating effects of adversity (Sperry & Widom, 2013) by facilitating more objective cognitive appraisals of the event, thus reducing harmful psychological and physiological responses and enhancing subjective expectation around coping (Dworkin et al., 2019). Conversely, unsupportive relationships may impede recovery and elevate psychological stress (Ehlers & Clark, 2000; Hansford & Jobson, 2022). For example, In a meta-analysis of 148 cross-sectional and 38 longitudinal effect sizes, Zalta et al. (2021) found a correlation between social support and post-traumatic stress severity (${R}_{cross= -.27;}$ ${R}_{long= -.25}$) indicating that social support is predictive of PTS severity and thus other stress related outcomes such as violence and aggression. In other words, where positive social supports exist, youth who have experienced violent trauma may be less likely to experience elevated stress and the associated debilitating effects of violent adversity (Sperry & Widom, 2013).

### The Unique Position of Youth Work to Provide Informal Social Support

Professional youth work is defined as the process of supporting the personal, social and educational development of young people across diverse settings (National Youth Agency, 2020). Youth workers are a valuable resource for sustainable social, emotional and behavioural change (Walsh & Harland, 2021), and in the context of complexity, holds the promise of saving lives (Thapar, 2021). In their study of youth work provision in England, UK Youth (2022) estimated that the indirect value of youth work exceeded £3bn, with more than £500 m in savings from reductions in crime alone. Socially targeted polices frequently cite the potential of youth work to contribute in a collective responses to issues such as interpersonal violence (Maxwell & Corliss, 2022), and yet the specific mechanisms of change are not well-established. A youth work methodology first and foremost is about a critical, relational-driven encounter with young people (Harland & McCready, 2012), wherein the youth worker meets young person on their own terms and endeavours to facilitate meaning-making from lived experiences. Professional youth work is in fact underpinned by learning environments that engage, stimulate and motivate young people, while also supporting them to explore their fears and aspirations and reflect on their experiences-good and bad (Jupp-Kina & Gonçalves, 2021). In the context of divided and violent societies such as Northern Ireland, those experiencing the greatest ecological stress are also those most at risk of marginalisation within communities (Harland & McCready, 2014). These youth are most likely to be affected by stress induced pathology (Sperry & Widom, 2013). Importantly, however, the literature suggests that the presence of positive social supports in one domain may dampen the risk of unsupportive social relationships in other domains (e.g., family, school and community) (Hansford & Jobson, 2022). Despite this, the association between social support, psychological stress and violence has been under-evaluated.

### The Present Study

Building upon previous empirical evidence that exposure to violent adversity is common, and that exposure to violence is implicated in the onset and maintenance of psychological stress, the aim of this study is to explore if social support operates through psychological stress to contribute towards violent outcomes. Building upon the previous literature, the following research questions were formulated:

1. *Is exposure to interpersonal adversity uniquely associated with elevated psychological stress in this sample of Northern Irish youth?*
2. *Is elevated psychological stress related to an elevated risk of physical violence?*
3. *Does social support operate through psychological stress to influence the risk of physical violence?*

## Method

### Participants

This study was part of a wider evaluation of the Engage project funded through the Northern Ireland Tackling Paramilitarism and Organised Crime Programme (DOJ, 2020). The sample included youth involved in that project. Demographic data is presented in Table 1.

**Table 1:** Participant characteristics (*N* = 635)

Characteristic		N/M	%
Gender	Male	454	71.5
	Female	164	25.8
	Prefer not to say	1	0.2
	Missing	13	2
Religion	CNR*	288	58.1
	PUL**	192	38.7
	Other	16	3.2
Age (M)		15.59 [10–25]	
NEET		175	39.4

*CNR = Catholic/Nationalist/Republication; **PUL = Protestant/Unionist/Loyalist

The majority of participants (*n* = 288, 58.1%) were from a Roman Catholic background, however, a significant minority were from a Protestant background (*n* = 192, 38.7%). Four participants (0.8%) self-designated as *‘other’* and 12 (2.4%) reported no affiliation to any religious group. The mean age across the sample was 15.59, ranging between ten years old and twenty-five years old.

### Data Collection

The baseline instrument was co-produced with the service providers and service users via four iterative stages. First, the author engaged with the providers to establish the primary and secondary outcomes for the programme. Second, the author engaged with the research evidence to establish a battery of suitable measures. Third, the author engaged with the service providers to assess acceptability and feasibility. Following minor amendments, the author engaged with a sample of youth participants to pilot the instrument and provide feedback. The author examined if the instrument could be completed, how long it took to complete and the young people’s perceptive on its completion. Once finalised, the instrument was available on an on-line platform (JotForm) to complete using phone, tablet or laptop. Youth workers were provided with a link to the baseline. Given the potential sensitivities, each young person completed their own baseline in confidence, using only a self-generated ID. This was unknown to either the researcher or the youth worker. On average, completion took eight minutes. Once completed, the responses were saved to a cloud-based server and exported for coding and analyses.

### Measures

#### Demographics

A series of demographic data including participant gender, age and educational/employment status was captured. In the context of Northern Ireland, another variable capturing politico-religious identity was also captured (see Table 1).

#### Trauma Checklist -Youth and Child

The Trauma Checklist is a twelve-item instrument that captures familial and community adversity. Additional questions to reflect the context of Northern Ireland (such as exposure to paramilitary related violence) were added to the items.

#### CRIES-8 (Perrin et al., 2005)

The CRIES-8 is a modified version of the Impact of Events Scale (Horowitz et al., 1979). to capture trauma related psychological distress. The measure consists of eight items designed to identify core PTSD symptoms of *‘re-experiencing’* and *‘avoidance’*. In several studies, the instrument has demonstrated both validity and reliability as a screening tool for PTSD with children aged eight years and above (e.g., Yule, 1997; Perrin et al., 2005; Morris et al., 2015; Duffy et al., 2021).

#### Likelihood of Violence and Offending Scale (Flewelling et al., 1993)

The eight-item scale is a short, self-report measure of violence. Each item is scored on a Likert scale ranging from 1–4 with options ranging from not likely at all (1) to very likely (4). For the purpose of this study, a new dichotomous variable was created using four items on the scale (physical fight, carry a gun, carry a weapon, injure someone) and coded as ‘1’ to reflect an intention to engage in any of these and ‘2’ little or no intention.

#### Oslo Social Support Scale (OSSS-3) (Kocalevent et al., 2018)

The OSSS-3 is a short, self-report scale of social support for use in the general population. With a Cronbach’s alpha *of* 0.640 the measure is acceptable given its brevity and economic structure. Following the broader literature, assessment of social supports can generally be considered in one of two ways: firstly, social support objectively offered and available, and secondly, social support that is perceived to be available (Dworkin et al., 2019). The three-item, one-factor structure of the OSSS-3 aggregates facets such as structural and instrumental support, and thus can be interpreted on a more generic level.

### Data Analysis

Descriptive and associational analyses were undertaken. To provide context, descriptive data illustrate the frequency of exposure to adversity, self-reported intent to engage in violence, expectation to be hurt in a violence related injury, as well a descriptive overview of the mean scores on the measure of psychological stress and social support. Subsequent bi-variate tests were undertaken to explore the differences in the mean scores on the measure of psychological stress and adversity and violence, as well as mean differences between those who expected to engage in violence and who did not on the measure of psychological stress.

To explore the hypothesis that social support operates through psychological stress to affect violent outcomes, a simple mediation model as conducted using the PROCESS macro (Hayes, 2017). The bootstrapping method was used to obtain 95% confidence intervals (95% CI) with 5000 re-samples for the indirect effects. Model 4 was conducted, entering social support as the independent variable, psychological distress as the mediator and self-reported violence as the outcome variable. Violent victimisation was entered as a covariate in the analysis. An effect was considered significant when the 95% CI did not contain zero.

## Results

### Is Exposure to Interpersonal Adversity Uniquely Associated with Elevated Psychological Stress in this Sample of Northern Irish Youth?

Participants were exposed to a range of interpersonal and non-interpersonal difficult life events, or adversities. 87.1% (*n* = 552) of the sample experienced at least one difficult life event (${M}^{2.15}$), ranging between no difficult life events for some and up to ten for others (SD = 1.74). 17.6% (*n* = 97) reported having been exposed to four or more distinct difficult life events. Interpersonal adversity, specifically violent adversity, was disaggregated by exposure to violence directly and indirectly in the home, in the community and a contextually relevant category-exposure to paramilitary related violence (see Table 2). 55.9% of the sample (*n* = 309) reported being exposed to at least one form of interpersonal adversity. Participants were most likely to witness community violence, but a significant proportion of the sample also reported direct victimisation at home as well as in the community.

**Table 2:** Exposure to violence

Violence context	N	%
Home (direct)	65	10.2
Home (witness)	76	12
Community (direct)	141	22.2
Community (witness)	236	37.2
Paramilitary threat	128	20.2
Paramilitary attack	35	5.5
Witness to paramilitary attack	212	33.4

Given the previous research findings that increased exposure to violence is associated with higher levels of post-traumatic stress, a screening tool for probable PTSD was administered. Scores ranged between 0 and 40 ($${M}^{Cries-8 }=9.92)$$. A total of 254 (40.1%) of those participants who completed the scale scored above the clinical cut-off ($$\ge$$ 17) for probable PTSD. Contextual as opposed to demographic factors were more strongly associated with this elevation. For example there was a significant dose–response effect of the number of difficult life events and elevated psychological stress, with a positive correlation between CRIES-8 scores and the total number of difficult life events reported by the participants (r = 0.55, *n* = 504, *p* =  < 0.001). With the exception of *‘another disaster’*, each form of adversity uniquely elevated stress as measured by CRIES-8 (see Table 3). However, participants who had experienced interpersonal, violent adversities (M = 14.94, SD = 11.61) were more likely to score higher on the measure of psychological stress than their peers without such experiences (M = 9.07, SD = 9.9; t (380) = 4.84, *p* =  < 0.001), a finding that is consistent with previous research.

**Table 3:** Relationship between CRIES-8 scores and life events

*Life event*	*N*	*m*	*SD*	*t*	*df*	*p*
Bad accident	70	19.03	9.8	5.61	123	< 0.001
	55	10.4	6.3			
Another disaster	11	15.64	9.69	1.79	70	0.078
	61	11.17	7.23			
War	15	22.2	13.77	2.94	15.98	0.01
	60	11.4	7.23			
Hit, punched or kicked hard at home	51	22.2	9.23	7.73	89.73	< 0.001
	55	10.99	6.57			
Seen a family member being hit, punched or kicked at home	66	20.53	10.48	7.64	98.74	< 0.001
	51	9.27	5.09			
Beaten up, shot or threatened by someone to be hurt	99	18.14	9.7	5.98	133.73	< 0.001
	48	10.21	6.23			
Witness community violence	170	16.46	9.3	8.16	119.87	< 0.001
	34	8.47	3.91			
Sexual violence	8	28.75	6.77	7.13	67	< 0.001
	61	10.67	6.74			
Heard about the violent death or serious injury of a loved one	116	17.28	9.8	4.95	93.22	< 0.001
	37	10.38	6.43			

### Is Elevated Psychological Stress Related to Elevated Risk of Physical Violence?

The data thus far supports the findings of previous studies and shows that for this sample of Northern Irish youth, greater exposure to violent adversity is associated with greater psychological stress. Another key objective of the current study was to understand if elevated stress translated into increased risk of violent victimisation and/or likelihood of perpetration. Interestingly, more than two-fifths of the sample (41.5%, *n* = 210) reported that they expected to be involved in physical violence within the next month. Further, half of the sample believed that they would be injured in the next month (51.9%, *n* = 252), and more than half believed that they would injure someone else (47.7%, *n* = 229). Elevated stress symptoms was associated greater risk of physical violence. Mean scores on CRIES-8 differed for those who reported an assumption of physical violence (m = 17.83, SD = 8.04) compared with those who did not expect to engage in physical violence in the next month (M = 12.95, SD = 8.03; t (322) = -4.7, *p* =  < 0.001).

### Does Social Support Operate through Psychological Stress to Influence the Risk of Physical Violence?

The majority of participants (71.8%; *n* = 321) indicated that there were adults in their lives which they admired. However, only 23.7% (*n* = 116) of the sample reported that accessing that social support was very easy. Further, 27% (*n* = 132) reported to have between one and two people that they could ‘count on’, with 5.7% (n = 28) reporting that they had nobody to count on for support. On the measure of social support, scores ranged between 1 and 14 ($OSS-{3}^{M} = 8.91$). 71.9% (*n* = 455) of the sample were within the *‘poor’* or *‘moderate’* social support banding, meaning that less than one-third of the sample were assessed as having *‘strong’* social supports (28%; *n* = 177).

**Fig. 1:**
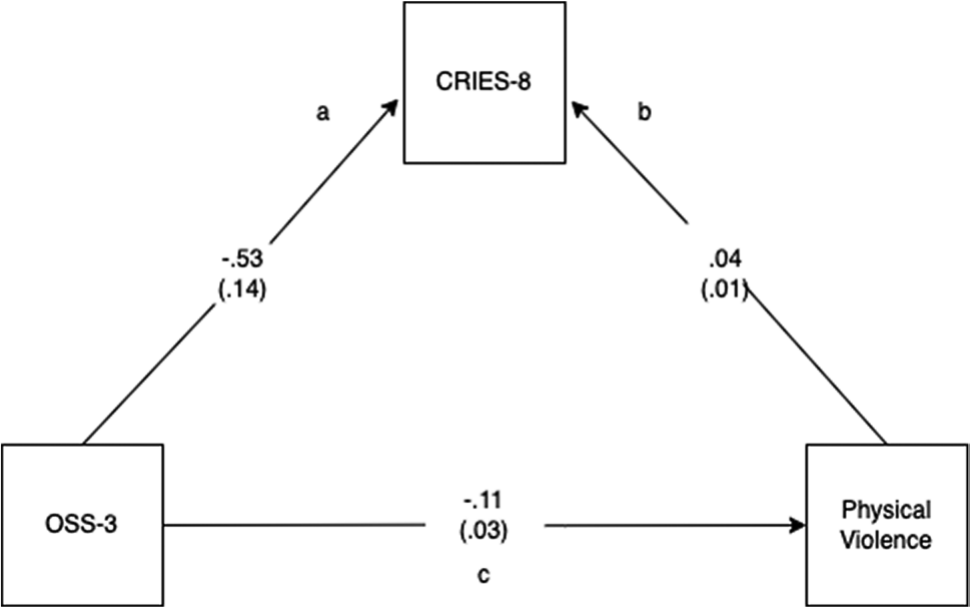
Mediation model (social support, psychological stress and violence)

To test the hypothesis that social support operates through psychological stress to increase the risk of violence, a simple mediation model was estimated using OLS path analysis to determine the effective of social support on intention to engage in violence through psychological stress, with exposure to interpersonal violence as a covariate. This model was calculated using PROCESS (Hayes, 2013) to estimate indirect effects to control for the variance explained by the mediator. 5000 bias-corrected bootstrap samples were used for the 95% confidence interval (see Fig. 1).

Regression models were tested to investigate whether social support operates through psychological stress. In the first model (path a), increased social support was significantly related to lower psychological stress (B = -5.3, SE = 0.14, *p* < 0.001), controlling for exposure to interpersonal violence. In the second logistic regression model, which included psychological stress and social supports as predictors of physical violence, both psychological stress (path b) (B = 0.04, SE = 0.01, *p* =  < 0.001) and social support (path c) B = -0.11, SE = 0.03, *p* =  < 0.001) were related to intention to engage in violence controlling for exposure to interpersonal violence. The indirect effect (path c’) was tested using a percentile bootstrap estimation approach with 5000 samples (Shrout & Bolger, 2002), implemented with the PROCESS macro version 3.5.3 (Hayes, 2017). The model (see Fig. 1) revealed that reduced social supports had a negative effect on intention to engage in violence (i.e., greater likelihood) through psychological stress. The bootstrap confidence intervals indicated that the total, indirect effect coefficient was significant, B = -0.02, SE = 0.008, 95% CI[-0.04, -0.006], which supported the hypothesis that controlling for psychological stress (CRIES-8), the effect of social support on intention to engage in physical violence in the next month was statistically significant.

## Discussion

In line with research into exposure to violence in conflict and high-crime areas, these youth were exposed to a range of ecological stressors, including violence in the home and violence in the community (Haj-Yahia et al., 2021; Walsh, 2021; Redican et al., 2022). Their communities, rather than a place of refuge, were often bastions of harm.

Whilst there has been growing interest in the frequency and intensity of youth violence, less has been made of its distribution. This study demonstrates that for some young people, and in clusters, violence is a pervasive adversity that is experienced across multiple environments (Nygaard et al., 2018; Redican et al., 2022; YEF, 2022). Indeed, when the experiences of these youth are compared with the general youth population, this sample experienced elevated rates of overall as well as specific adversity. Compared with 37% of the general Northern Ireland youth population, 87.1% (n = 552) of this sample had experienced at least one difficult life event (Bunting et al., 2020). Over half of the youth were exposed to interpersonal adversities (Leenarts et al., 2013), with violence highly prevalent across the sample (Bunting et al., 2020). Both have been implicated in negative psychological outcomes (Finkelor et al., 2007; Brady et al., 2008; Nöthling et al., 2019; Hamby et al., 2021; Zalta et al., 2021). Compared with an estimate of 2% in the general youth population (Bunting et al., 2020), 40.1% (*n* = 254) of this sample scored above the cut-off for probable PTSD, indicating that those involved in this study represented a cluster of youth most exposed to ecological stressors and suffering most from its stress inducing effects (Sperry & Widom, 2013). Violence and its harms reflect the lived reality for many youth (Walsh & Gray, 2021) and thus these observations should have practical implications. Failing to acknowledge these potentially traumatic experiences and subsequently failing to respond to the needs of traumatised youth may serve to normalise violence and aggression and impede recovery and normative developmental processes.

In line with previous research, this study found that there was a dose-response effect of exposure to adversity (Finkelhor, 2018; Malvaso et al., 2022), with youth who had experienced more difficult life events, more likely to screen positively for probable PTSD (Duffy et al., 2021). From previous research, we know that a relationship exists between psychological stress and violent behaviour (Fowler et al., 2009; Losel & Farrington, 2012). This study supports those previous observations and found that elevated psychological stress was associated with youth reporting an intention to engage in violence (Widom, 1989; Sperry & Widom, 2013; Wright et al., 2019; Zalta et al., 2021), but also points towards prevention.

There is concern that the therapeutic needs of victims of violent trauma often go unrecognised often resulting in long-term consequences for the individuals as well as the wider community (Teicher et al., 2016). Research on the role of social support during childhood, and the onset of psychopathology and violence is sparse (Sperry & Widom, 2013). Despite the potential for traumatic responses following exposure to violence (Cloitre et al., 2009; Finkelhor, 2018; Malvaso et al., 2022), the role of social support as a buffer has been under-evaluated, particularly with young people (Brewin et al., 2000; Ozer et al., 2003; Blais et al., 2021). The findings from this study suggest that even in contexts of pervasive threat and violence, young people who are at elevated risk of psychological distress and further violence can be protected via the mechanism of positive social supports (Guay et al., 2006; Maschi & Bradley, 2008). This study has shown that social supports operates through psychological stress and therefore may contribute towards the goal of violence reduction (Trickey et al., 2010; Sperry & Widom, 2013; Zalta et al., 2021). For youth vulnerable to violent trauma, the youth work approach is uniquely positioned to engage with those most at risk and marginalized in communities (Walsh & Harland, 2021; Thapar, 2021) where violence and its related harms are clustered. This study adds to the literature by highlighting the potential utility of social support focussed interventions in the field of violence prevention (Zalta et al., 2021) and provides an evidence base for the role of youth work which has at its core, relational-driven encounters (Harland & McCready, 2014).

In summary, this study found that exposure to interpersonal adversity was uniquely associated with elevated psychological stress, controlling for violent victimisation; that elevated psychological stress was related to elevated risk of physical violence and; social support operates through psychological stress to influence the risk of physical violence. Whilst there is a risk of type I error (false positive) associated with exploratory studies such as this that do not adjust for multiple testing, combined, these insights illustrate one of the valuable characteristics of youth work provision for youth vulnerable to violent adversity and provide opportunities for harm reduction and prevention.

## Limitations and Future Directions

Despite the value added through this study, a number of limitations are acknowledged. Firstly, social support does not negate the need for clinical assessment and evidence-based treatment. Youth who are most traumatised may require specialist supports from trained clinicians (Duffy et al., 2021). That said, where social support is enhanced, opportunities to connect those most in need of psychological treatment may be more likely. Secondly, this study does not elucidate the conditions nor the mechanisms that facilitate effective social support. There is a need to understand these mechanisms, as well as the competencies of youth workers that may remediate psychological stress and reduce the risk of violence. Thirdly, this study was unable differentiate between perceived social support and the nature of social support received (Dworkin et al., 2019). For instance, whilst support may be available, it may not be interpreted as such by youth in need of it. This would be useful to capture in future studies. Lastly, evidence suggests that the contexts in which social support is provided may be stronger predictors of improved psycho-social functioning (Nygaard et al., 2018). For example, there may be a difference in general social support routinely available and social support provided in the context of trauma disclosures. The latter has been implicated in more negative outcomes, particularly when disclosures are met with a negative response (Sperry & Widom, 2013). Capturing such contexts would add value to our understanding of when, as well as how social support may alleviate the harmful psychological and physiological responses to violent trauma and enhance coping (Dworkin et al., 2019).
